# Molecular docking based screening of Listeriolysin-O for improved inhibitors

**DOI:** 10.6026/97320630013160

**Published:** 2017-05-31

**Authors:** Sara Ghafari, Matin Komeilian, Mohaddese sadat Hashemi, Sareh Oushani, Garshasb Rigi, Behnam Rashidieh, Kamran Yarahmadi, Fatemeh Khoddam

**Affiliations:** 1 Vira Vigene research institute, Tehran, Iran; 2 Department of Biology, Faculty of Science, Behbahan Khatam Alanbia University of Technology, Behbahan, Iran

**Keywords:** Listeria monocytogenes, Listeriolysine-O, Molecular docking, Drug discovery

## Abstract

Listeriolysine-O (LLO) is a 50KDa protein responsible for Listeria monocytogenes pathogenicity. The structure of LLO (PDB ID: 4CDB) with domains D1 to D4 is known. Therefore, it is of interest to identify conserved regions among LLO variants for destabilizing oligomerization (50 mer complex) of its monomers using appropriate inhibitors. Therefore, it is of interest to identify suitable inhibitors for inhibiting LLO. Previous reports suggest the use of flavanoids like compounds for inhibiting LLO. Our interest is to identify improved compounds to destabilize LLO oligomerization. We used a library (Zinc database) containing 200,000 drug-like compounds against LLO using molecular docking based screening. This resulted in five hits that were further analyzed for pharmacological properties. The hit #1 (2-methyloctadecane- 1, 3, 4-triol) was further refined using appropriate modifications for creating a suitable pharmacophore model LLO inhibition. The modified compound (1-(4-Cyclopent-3-enyl-6, 7-dihydroxy-8-hydroxymethyl-nona-2, 8-dienylideneamino)-penta-1,4-dien-3-one) shows fitting binding properties with LLO with no undesirable pharmacological properties such as toxicity.

## Background

Listeria monocytogenes is known for its virulence among the most famous species of Listeria [1]. Nevertheless this gram-positive bacterium is a facultative intracellular pathogen; the disease might be occurred due to germ entrance through contaminated foods or beverages into a healthy body [2]. While this bacterium is to some extent resistant to Gastric acid and Bile salts [3], one of the L.monocytogenes's toxin proteins so called: Listeriolysine-O (LLO) is contributing to the pathogenicity of the organism and enables it to achieve its fatality especially in pregnant women following to invade the tissues of the host thus it is known as a virulence factor [4,5]. Particularly the hallmarks of this factor are to be nonenzymatic, cytolytic, thiol-activated, cholesterol-dependent, poreforming toxin and most notably it remains in an active form even after bacteria׳s death [6]. It also can induce cytolysis in infected host cells even in low concentration of 5 ng/ml [7]. Furthermore, LLO mediated cell death is proceeded through cytolysis or apoptosis [8]. Apoptotic event, which is mediated by LLO, can induce two pathways: mediating by activation of caspase-3 and caspase-6, another pathway dependent on LLO but independent to caspase [9]. First pathway in apoptotic T-cells have been described. Activation of mechanism by 3 surface receptors starts. Activated Tcells have obtained FAS L as ligand receptors. Attachment of LLO as a ligand to FAS L leads to activation of death domain, which attaches to the outer domain. Death domain also plays a pivotal role in connecting caspase as an adaptor, so this interaction can activate pro-caspase. This factor realizes caspases-3 that can inactivate DNA. All these factors end in segmenting DNA in the host cell [10].

Koster et al have revealed Crystal structure of LLO in in 2014. According to the crystal structure, it consists of four distinct domains, which have been called D1 to D4 and each of them, playing a different role in LLO functionally. This study was conducted to intend to illustrate some sequences of LLO, which are more conserved in D1 to D4. Describing these sequences can be of a great importance for modeling an inhibitor for inhibiting oligomerization of LLO monomers [11]. Two conserved sequences in D1 can be noted. Firstly, the sequence contained 25 amino acids, which are significant for LLO function. Additionally, LLO has another important region that is known as a PEST-like sequence (P: proline, E: glutamine, S: serine,T: theronine). This region is essential for virulence and L. monocytogenes's in-vivo function [12]. Moreover, 91-99 sequences of D2 play a key role in immunogenic and identified by CD+8 T-cells but a highly conserved motif structure in D4 consists of 11 residues and makes contribution to cytotoxicity of L. monocytogenes. LLO is able to connect to the intestinal epithelial by Internalin protein. Also, LLO is one of those factors, which released by infected cell to other cells. Pore forming is a mechanism that causes transition and during this procedure a hole is created in host cell resulted in cytolysis in the host cells and finally leads to cell death [13].

During past these years, some drug targets were introduced with bactericidal mechanism: Yasuhiro Gotoh et al. (2010), worked on specific inhibitor against two component signal transduction systems (TCSs) which could reduce virulence of bacteria with inhibiting the sensory domains of the sensors blocking the quorum sensing system [14]. One year later, Mikael Mansjo along with Jorgen Johansson introduced FMN riboswitch as a novel drug target for antibacterial substances. They investigated how flavine analog, roseoflavin, affected the growth and infectivity of L.monocytogenes at a very low concentration. Interestingly, their results admitted that roseoflavin enhances L.monocytogenes virulence in mechanism independent of the FMN riboswitch [15]. Wang J et al. also showed the detection of LLO native inhibitors with contrasting activity by using hemolysis test [16] but the inhibitors should be improved. LLO is pore forming toxin (PFTs) and its monomers oligomerize into ring of 50 monomers. Accordingly this virtual experiment, such as our other studies in this field [17,18,19], is conducted to aim for modeling an essential inhibitor for prohibiting oligomerization of LLO monomers, which causes to induce inhibiting of oligomer formation and prevent pore forming of LLO [20,21,22,23].

## Methodology

### Protein and ligand structures

The X-ray crystal structure of 488 amino acids polymer of L. monocytogenes toxin (LLO) obtained from protein data bank (www.rcsb.org/pdb/home/home.do) with pdb access code of 4CDB [11]. The provided structure was a pore containing up to 50 monomers with a diameter of 300 Å. Considering the aim of this study that is to inhibit monomer interactions, the chain A of this Alpha-helical Pore-forming toxin was separated as the target for drug binding. Besides, to provide biological conditions simulation, the monomer structure was solvated in a water box with the distance of 2 Å. Adding Na or Cl ions then neutralized the system. The solvation process was carried out by GROMACS 4.5.6 simulation software. Zinc database (http://zinc.docking.org/) was used as the ligand database for virtual screening [24]. A subset from drug-like category obtained and used as virtual screening library. Virtual screening was performed among 200,000 ligands and top successive hits were selected for rational drug design purpose.

### Virtual screening

PyRX software [25] was used as the virtual screening software. PyRX includes Autodock vina [26] with a Lamarckian genetic algorithm as scoring algorithm.

### Pharmacokinetic analysis and rational drug design

FAF Drugs 3 web server [27] was used to analyze the absorption, distribution, and metabolism properties of top 10 virtual screening hits. Moreover, the toxicity properties were analyzed using the PROTOX web server [28]. New ligands were designed based on the structure of top hits retrieved from virtual screening process. To achieve this goal, hyperchem software was used. New rationally designed ligands were then analyzed regarding ADME and toxicity to reach optimal scores.

## Results and Discussion

**Figure 1:**
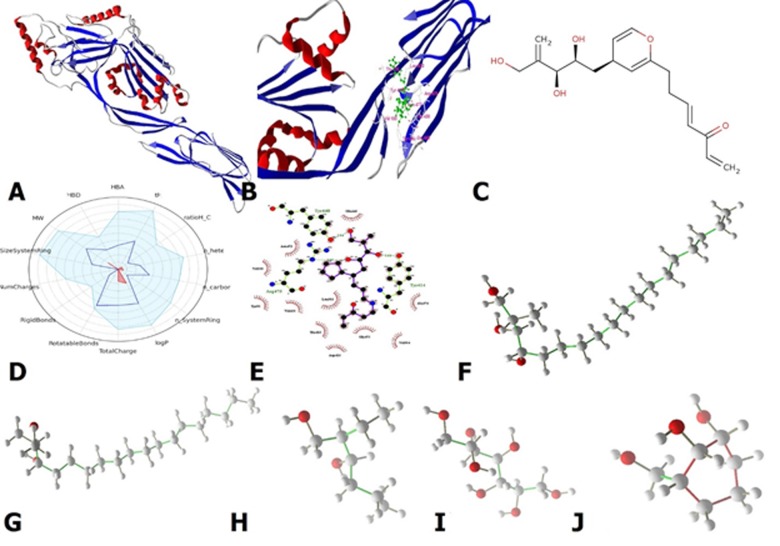
A: The structure of LLO, it consists of four distinct domains, which have been called D1 to D4 and each of them, playing a different role in LLO functionally. B: The modified structure (RD-1) in contact with D2 domain of LLO. C: The unbound RD-1 structure. D: The overall pharmacological properties of RD-1. E: The ligand map of RD-1. F: The structure of Hit#1. G: The structure of Hit#2. H: The structure of Hit#3. I: The structure of Hit#4. J: The structure of Hit#5

Among 200.000 drug-like ligands, the 5 highest binding affinity hits were selected for the further evaluation. These top 5 poses, which indicated more negative binding affinity, were examined for pharmacokinetic analysis and rational drug design purpose. The selected hits reached the binding affinity equal to -9.6 for hit 1, -9.4 for hit 2, -9.1, -8.8 and -8.6 for hit 3, 4 and 5 respectively. Based on Lipinski rule of 5 we analyzed the hits regarding pharmacological properties. In Table 1 the pharmacological properties of these top hits are presented. To reach the best inhibitor, we focused on PEST sequence in D1 of LLO structure, which is close to the N-Terminus. This sequence plays a key role in LLO dependent bacterial virulence. Hit 1 and hit 2 are very similar in structure just with one hydroxyl group differentiation. Other hits are very small so they seemed to be not suitable for being candidate as this limitation prevented protein-protein interaction correctly. For acquiring the specific binding affinity we tried to design specific ligands, which could tightly bind to D1 domain. To do this, we chose hit 1 because of high binding affinity and appropriate molecular weight. Thus, initially we endeavor to modify hit 1 to reach its optimal pharmacological properties. Based on FAFDrugs3 output, there are some errors in rotatable bonds and LogP. So based on hit 1, RD-1 was rationally designed and to decrease LogP, one carbonyl was added to the primary structure. Also in the base structure, there were problems in rotatable bonds. Changing rotatable bonds directly affects the binding affinity. Several refinement steps were applied in the base structure to decrease its rotatable bonds in a manner that the binding affinity remains still high. The propane group was removed from the structure end to implement this change and also five double bonds were added to solve the rotatable bonds problem. These structurally modifications and substitutions were changed the pharmacophore model seriously but the binding affinity still was -9.2. The new structure was checked again and still the same error was exits. To solve this problem, we have added a cyclopentane substitute and nitrogen was added in center of RD-1. These refinements were not significant effected other parameter such as complexity and tPSA. After these changes, RD-1 finally passed FAFDrugs successfully with the binding affinity equal to -9.1. The final structure of RD-1 in contact with LLO following its ligand map is depicted in Figure 1.

In order to calculate the LD50 and probable nonspecific targets, PROTOX webserver was used. PROTOX prediction indicated LD50 of RD-1 is 1170 mg/kg with toxicity class 4. This performance operated in average similarity 80.26% and prediction accuracy of 78.26 %. Interestingly no toxicity target (nonspecific) was found by PROTOX as well. As FAFDrugs and PROTOX indicated, the RD-1 is a lead compound and can serve as a new drug to inhibit LLO.

## Conclusion

Identification of an LLO inhibitor to destabilize its 50-mer oligomerization is of interest. We describe the identification, modification and design of an LLO inhibitor named RD-1 with fitting properties for further consideration. It should be noted that further in vitro studies are needed to confirm binding and inhibition of LLO.

## Figures and Tables

**Table 1:** The pharmacological properties of the top hits

Ligand No	Compound	MB	HBdonar	HBa	Solubility Mg/l	Rotatable bond	Rigid Bond	Stero centres	Binding affinity
1	2-methyl-octadecane-1,3,4-triol	316.52	3	3	2816.98	16	0	3	-9.6
2	2-methyl-octadecane-1,3 -diol	300.52	2	2	1422.18	16	0	2	-9.4
3	2-ethyl-hexane 1,3-diol	146.23	2	2	41706.33	5	0	2	-9.1
4	heptane 1,2,3,4,5,6,7 -heptaol	212.2	7	7	1426233.86	6	0	5	-8.8
5	3-hydroxymethyl-cyclohexane-1,2-diol	146.18	3	3	75923.44	1	6	3	-8.1
RD-1	1-(4-Cyclopent-3-enyl-6, 7-dihydroxy-8-hydroxymethyl-nona-2, 8-dienylideneamino)-penta-1,4-dien-3-one	345.43	3	5	41573.02	11	11	4	-9.1
